# Long read genome assemblers struggle with small plasmids

**DOI:** 10.1099/mgen.0.001024

**Published:** 2023-05-24

**Authors:** Jared Johnson, Marty Soehnlen, Heather M. Blankenship

**Affiliations:** ^1^​ Michigan Department of Health and Human Services, Bureau of Laboratories, Lansing, MI, 48906, USA

**Keywords:** Plasmids, whole-genome sequencing, long-read sequencing, hybrid genome assembly

## Abstract

Whole-genome sequencing has become a preferred method for studying bacterial plasmids, as it is generally assumed to capture the entire genome. However, long-read genome assemblers have been shown to sometimes miss plasmid sequences – an issue that has been associated with plasmid size. The purpose of this study was to investigate the relationship between plasmid size and plasmid recovery by the long-read-only assemblers, Flye, Raven, Miniasm, and Canu. This was accomplished by determining the number of times each assembler successfully recovered 33 plasmids, ranging from 1919 to 194 062 bp in size and belonging to 14 bacterial isolates from six bacterial genera, using Oxford Nanopore long reads. These results were additionally compared to plasmid recovery rates by the short-read-first assembler, Unicycler, using both Oxford Nanopore long reads and Illumina short reads. Results from this study indicate that Canu, Flye, Miniasm, and Raven are prone to missing plasmid sequences, whereas Unicycler was successful at recovering 100 % of plasmid sequences. Excluding Canu, most plasmid loss by long-read-only assemblers was due to failure to recover plasmids smaller than 10 kb. As such, it is recommended that Unicycler be used to increase the likelihood of plasmid recovery during bacterial genome assembly.

## Data Summary

The authors confirm all supporting data, code and protocols have been provided within the article or through supplementary data files. All sequence data used in this study can be found under bioprojects PRJNA929966, PRJNA288601, and PRJNA812922. Additional supporting code and supplementary data can be found at https://github.com/johnsonj161/for_publications/tree/107c3dd3865cff8ab5bd86ef70249a65b465aa9c/Long%20read%20genome%20assemblers%20struggle%20with%20small%20plasmids.

**Impact Statement**

Plasmids often play important roles in bacterial evolution and public health. As such, it is essential that we capture plasmid sequences when conducting whole-genome sequence analysis. Long-read genome assemblers have been reported to sometimes miss plasmid sequences, particularly those that are smaller in size. Results from this study confirm these prior reports, demonstrating that many long-read assemblers are prone to missing plasmids smaller than 10 kb. Further, we demonstrate that the short-read-first assembler, Unicycler, was able to recover all these plasmids when using a combination of Illumina and Oxford Nanopore reads. Therefore, we recommend that Unicycler be used instead of long-read-only assemblers, when the recovery of plasmid sequences is important.

## Introduction

Plasmids are extrachromosomal genetic elements found in bacteria that often play important roles in bacterial evolution and public health [1]. Like the chromosome, plasmids are generally circular and can harbour important virulence genes but differ in that they are normally smaller (744 bp to 2.58 Mb) and can be shared through horizontal gene transfer [2]. Further, a bacterium can harbour multiple different plasmids and/or multiple copies of the same plasmid in a single cell [1].

High throughput sequencing (HTS) has become a preferred method for studying bacterial genomes and plasmids, as this approach is generally thought to capture the entire bacterial genome [3]. However, the quality of plasmid assemblies generated from HTS data can greatly depend on the sequencing technology used. Assemblies generated from Illumina short-read sequences have a low error rate but are often highly fragmented, thus making it difficult to confidently differentiate plasmidic sequences from chromosomal sequences. By contrast, long-read sequencing technologies, Oxford Nanopore Technologies (ONT) and PacBio, often produce assemblies that are structurally complete but sometimes miss plasmids due to biases introduced during library preparation [4, 5]; and/or issues with long-read genome assemblers [6–8]. To overcome these issues, a hybrid approach can be employed, which leverages the strengths of both long- and short-read sequencing technologies, to produce assemblies that are highly accurate and structurally complete [4, 9].

Early hybrid genome assembly methods, like Unicycler, utilize a short-read-first approach, where contigs constructed from short reads are used as scaffolds during long read assembly [10]. This approach has since been suggested to be replaced with a long-read-only approach, where long reads are used to build the initial genome assembly, which is then polished using the high-quality short reads [7]. While this long-read-only approach has been demonstrated to produce high quality assemblies, its reliance on long-read assemblers to produce the initial assembly means it is also likely to miss plasmid sequences [4].

Among long-read genome assemblers, Canu and Flye are often reported to perform best in terms of overall genome quality, completeness, and plasmid recovery; however, benchmarking studies still report these assemblers are not capable of capturing 100 % of plasmid sequences [6, 11]. Since these studies, Flye has been updated to improve how plasmid sequences are handled, including a ‘metagenomics’ option (--meta) (released 2.8; 4 August 2020; https://github.com/fenderglass/Flye), which is meant to improve the assembly of contigs with uneven sequence depths – a situation often experienced with plasmid sequences that are present at high copy numbers in a single cell. The Flye 2.9 update also introduced a new high accuracy mode (--nano-hq) (release 2.9; 20 August 2021) that is meant to handle long read sequences with improved (3–5 %) basecalling error rates.

Some evidence suggests that the ability of long-read assemblers to recover plasmid sequences could be related to the size of the plasmid [6, 11] however, this relationship has not been thoroughly explored. As such, the goal of this work was to investigate the relationship between plasmid recovery by common long-read assemblers and plasmid size, while also testing if the Flye v2.9 updates improve plasmid recovery, as compared to earlier versions used in previous benchmarking studies. Plasmid recovery rates by long-read sequencers were additionally compared to the recovery rates by the short-read-first assembler, Unicycler. This was accomplished by determining the number of times each assembler successfully recovered 33 plasmid sequences from 14 complete bacterial assemblies, belonging to six species, generated using the Trycycler method with Oxford Nanopore long reads and/or Illumina short reads.

## Methods

### Bacterial culturing, DNA extraction, and sequencing

Bacteria used in this study are listed in Table 1. Isolates were obtained from either the American Type Culture Collection (ATCC) or from the Michigan Department of Health and Human Services (MDHHS). ATCC strains were revived from lyophilized cultures following ATCC recommendations. All MDHHS isolates were from clinical origins. Freezer stocks of all isolates were made from single colonies grown for 24 to 48 h on Blood or Chocolate agar (Thermo Scientific, Waltham, MA) at 37 °C. DNA was extracted following the CDC PulseNet Total DNA Extraction method (PNL33) from colonies grown from freezer stocks for 24 to 48 h at 37 °C on Blood or Chocolate agar. DNA concentration was determined using Qubit dsDNA Broad Range assay (Thermo Fisher, Waltham, MA). Paired-end short read libraries were created using the Illumina DNA Prep kit (Illumina, San Diego, CA) with Nextera CD DNA indices and sequenced on a MiSeq using the Illumina v3, 600 cycle kit. Long read libraries were created using the ONT Rapid Barcoding kit (SQK-RBKP004) (ONT, Oxford, UK) and sequenced on an Mk1C using R9.4.1 flowcells.

**Table 1.** Summary of bacteria used in this study and their associated small (< 10 kb), medium (10 kb – 99 kb), and large (≥ 100 kb) plasmids

		**Plasmids (bp)**		
**Species**	**Strain**	**Large** (**≥ 100 kb**)	**Medium** (**10 kb – 99 kb**)	**Small** (**> 10 kb**)
* Escherichia coli *	ATCC 25922	–	93832, 24 185	3173, 1919
	2021QW-00057	118 339	46 161	4715, 4084, 4063, 2101
* Klebsiella pneumoniae *	2020QW-00078	194 062	97090, 34 331	–
	ATCC BAA-2146	140825, 117 755	85 160	2014
	2021QW-00045	190 147	97 090	–
	2021QW-00056	–	95985, 12 268	–
* Neisseria gonorrhoeae *	2022 NG-0076	–	–	4207
	2022 NG-0032	–	–	4153
* Salmonella typhimurium *	ATCC 14028	–	93 832	–
* Listeria innocua *	ATCC 33090	104 612	–	–
* Staphylococcus aureus *	ATCC 14458	–	30891, 15 773	4439, 4326
	ATCC 23235	–	27269	–
	2022QW-00133	–	43 879	–
	ATCC 25923	–	27490, 27 080	–

### Long-read, short- read, and hybrid genome assembly

Illumina short reads were trimmed using fastp v0.220 [12] and then quality checked using FastQC v0.11.9 [13]. ONT long reads were first processed using fast basecalling in MinKnow v21.11 (min qscore=8) and then re-basecalled with a high-accuracy model (dna_r9.4.1_450bps_hac) using Guppy v6.4.2+97a7 f06 (min qscore=8). High accuracy long-reads were then split into three subsets using the ‘subsample’ function in Trycycler v0.5.3 [7]. Draft genome assemblies of each isolate were created in triplicate from these subsets, using long read genome assemblers, Flye v2.9-b1768 [14], Miniasm v0.3-r179 [15], Raven v1.5.1 [16], and Canu v2.2 [17], and the short-read-first hybrid genome assembler, Unicycler v0.4.8 [10]. Unicycler assemblies were created using default settings with the full Illumina short read dataset and each of the ONT subsets. Flye assemblies were created using the ‘--nano-raw’, ‘--nano-hq’, and ‘--nano-hq + --meta’ options, herein referred to as Flye-raw, Flye-hq, and Flye-meta assemblies, respectively. Miniasm assemblies were created from alignment files generated by Minimap2 v2.23-r1117-dirty using default settings and then polished using Minipolish v0.1.3 [11]. Raven assemblies were created using default settings. Canu assemblies were generated using the ‘-fast’ option.

Completed assemblies of each isolate were additionally created using replicate Flye-meta, Raven, Miniasm, and Unicycler draft assemblies (n_total_=12), following the Trycycler method [7]. Replicate assemblies were clustered and the most likely contig combinations were selected based on 1) their overall support by each assembler, 2) support for existing contigs in the NCBI database, as determined using BLASTn v2.12.0 [18], 3) pairwise length and Mash [19] distances, as determined by Trycycler, and 4) evidence of contig circularization by Unicycler or Flye. Contig clusters were discarded if they contained two or fewer contigs or showed evidence of being fragmented pieces of a larger, circularized contig. Consensus sequences were generated from the selected clusters and polished three times with Medaka v1.6.1 (long reads) (ONT, 2022) [20] and then three times with Polypolish v0.5.0 [21] (short reads). The completeness and quality of each assembly was evaluated using CheckM v1.2.0 [22] and Quast v5.0.2 [23]. The recovery of all plasmids in the complete assemblies was supported by searching for evidence of plasmid sequences in the unused long and short reads. This was accomplished by mapping the reads back to the reference assemblies and searching for evidence of plasmids in the unmapped reads using PlasmidFinder v2.1.6 [24].

### Determination of plasmid sequence recovery and misassemblies

Plasmid sequence recovery rates for each draft assembly were determined by aligning the draft assembly to the respective completed assembly, using Minimap2 with the ‘--paf-no-hit’ option. Plasmid sequences were considered present if the total draft assembly alignment length exceeded 90 % of the reference contig length. In the case that more than one draft contig aligned to a reference contig, the total length of all aligned draft contigs was considered. It was noticed during the Trycycler process that some assemblers would erroneously assemble the plasmid sequences in the chromosome or create multiple copies of a plasmid sequence within a single draft assembly. For this reason, draft plasmid sequences were further classified as misassemblies if the plasmid sequence was found in the draft chromosome or if multiple contigs from a single draft assembly aligned to >90 % of the same reference plasmid.

## Results and discussion

### Sequence data and hybrid genome assembly quality

**Fig. 1.**
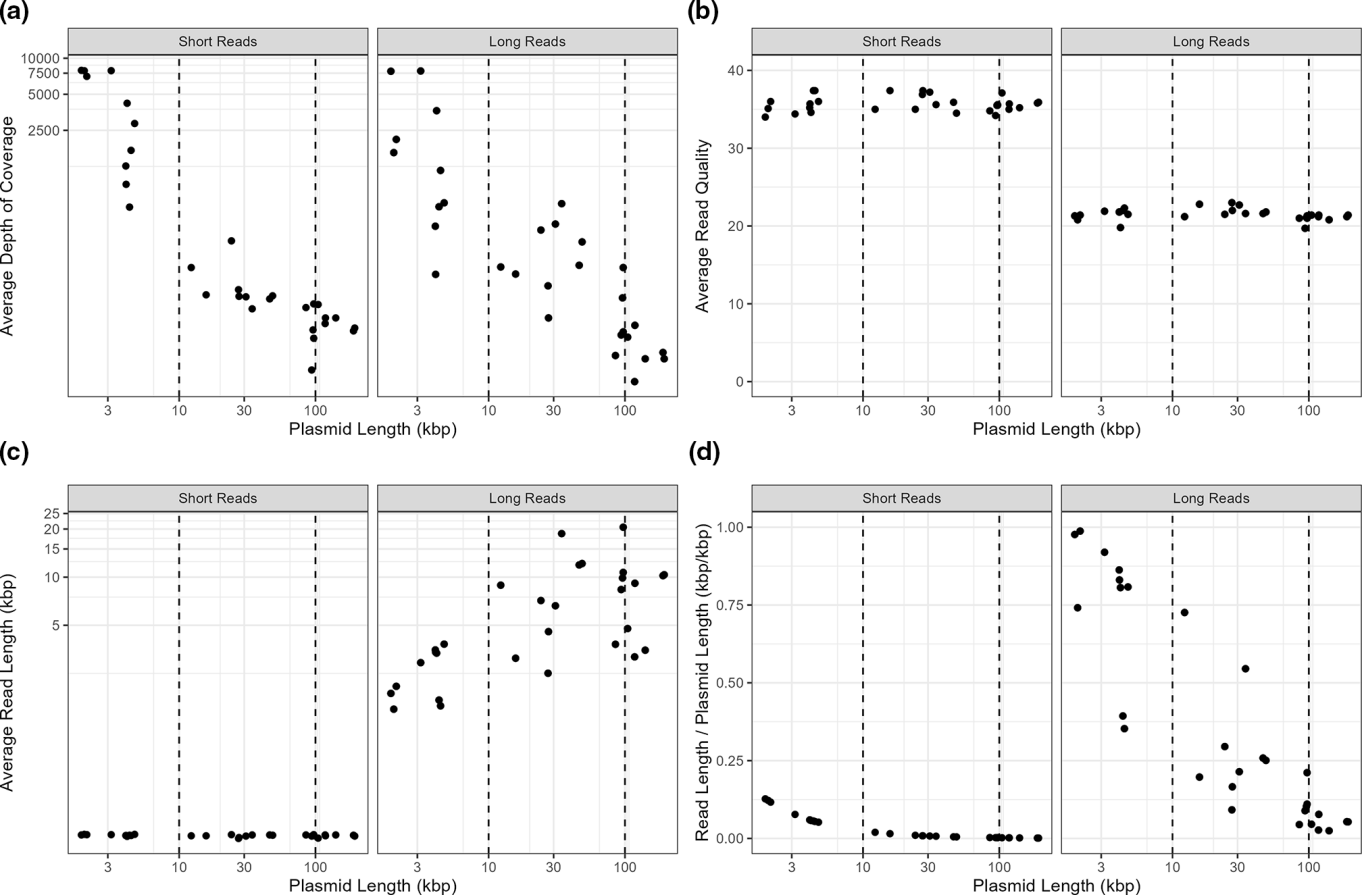
The relationship between contig length and read quality metrics, including (a) average depth of coverage, (**b**) average read quality, (**c**) average read length, and (d) the ratio between the average read length and the length of the associated plasmid. Dashed vertical lines represent the separation of small (< 10 kb), medium (10 kb – 99 kb), and large (≥ 100 kb) plasmids.

All hybrid assemblies generated using the Trycycler method contained fully resolved (i.e. circularized) contigs and were predicted to be >99.3 % complete by CheckM (Table S1, available in the online version of this article), with no evidence of missing plasmids via PlasmidFinder. Average lengths of the trimmed, high accuracy ONT reads ranged between 2566 to 10 815 bp (Table S1). ONT sequencing has been demonstrated to under-represent plasmid sequences depending on the library preparation kit used [5]. In agreement, the rapid barcoding kit used in this study was highly successful at recovering plasmid sequences, resulting in roughly 1-, 5-, and 50-times greater depth of coverage for the large, medium, and small plasmids, as compared to the chromosome (Fig. 1a). These differences in sequencing depth were also reflected in the short read sequences (Fig. 1a), thus indicating that these plasmids were likely present in the cell as multiple copies. Average read length also scaled with plasmid size, with reads associated with small plasmids often spanning >75 % of the total plasmid length (Fig. 1c, d). This contrasted with read quality (Fig. 1b), which did not display any notable relationship with contig size and read length for either read type.

### Plasmid recovery rates

**Fig. 2.**
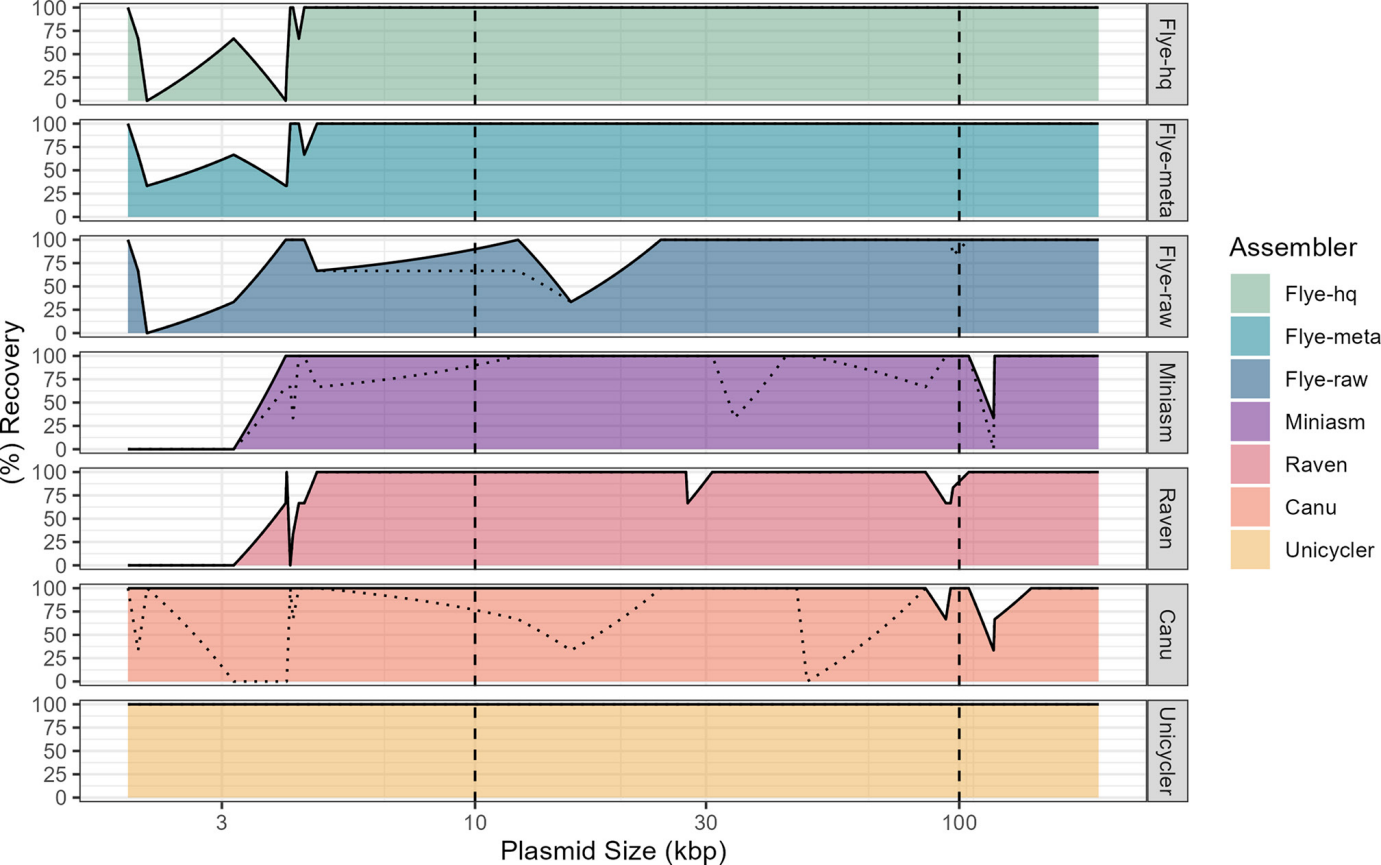
Area plots showing the relationship between plasmid size and plasmid recovery for long- and short-read-first assemblers, Flye, Miniasm, Raven, Canu, and Unicycler. Flye assemblies were generated using the ‘--nano-hq’, ‘--nano-hq + --meta’, or ‘--nano-raw’ options. Unicycler assemblies were generated using both long and short reads. Solid lines represent plasmid recovery rates when misassemblies are not considered. Dotted lines represent recovery rates when misassemblies are considered. Dashed vertical lines represent the separation of small (< 10 kb), medium (10kb – 99 kb), and large (≥ 100 kb) plasmids.

Altogether, 33 plasmids were evaluated, including six large (**≥** 100 kb), 16 medium (99 kb to 10 kb), and 11 small (< 10 kb) plasmids, with each assembly having one to six plasmids (Table 1). All plasmids were determined to be circular. Plasmid recovery rates differed greatly depending on the assembler used, the length of the plasmid, the species, and if misassemblies were considered (Table 2; Fig. 2). Unicycler recovered 100 % of plasmid sequences in all replicate assemblies. By contrast, Canu recovered 96 % of plasmids in replicate assemblies, followed by Flye-raw (91 %), Flye-meta (90 %), Flye-hq (88 %), Miniasm (85 %), and Raven (75 %) (Table 2; Fig. 2). These findings support previous benchmarking studies which found that Canu and Flye perform best among long read assemblers, in terms of plasmid recovery [6, 11] but indicate that even with the Flye 2.9 update, continued improvements to both assemblers are needed to ensure that all plasmid sequences are recovered.

**Table 2.** Recovery rates of small (< 10 kb), medium (10 kb – 99 kb), and large (≥ 100 kb) bacterial plasmids by long- and short-reads-first genome assemblers in replicate genome assemblies (*n*=3). The total percent plasmid recovery is shown, along with the percentage of recovered plasmids that were erroneously identified in the chromosome or present as multiple copies in a single assembly

		**% Plasmids Recovered**		
**Plasmid Size**	**Assembler**	**Total**	**In Chromsome**	**Multiplicated**
Large (≥ 100 kb) (*n*=18)	Flye-hq	100	0	0
	Flye-meta	100	0	0
	Flye-raw	100	0	0
	Miniasm	89	6	0
	Raven	100	0	0
	Canu	83	0	0
	Unicycler	100	0	0
Medium (99 kb – 10 kb) (*n*=48)	Flye-hq	100	0	0
	Flye-meta	100	0	0
	Flye-raw	96	0	4
	Miniasm	100	2	4
	Raven	92	0	0
	Canu	98	0	13
	Unicycler	100	0	0
Small (> 10 kb) (*n*=33)	Flye-hq	67	0	0
	Flye-meta	73	0	0
	Flye-raw	79	0	0
	Miniasm	64	0	29
	Raven	39	0	0
	Canu	100	0	36
	Unicycler	100	0	0

Small plasmids (< 10 kb) were missed most frequently by long-read-only assemblers (Table 2; Fig. 2), with all plasmids in this category being missed by at least one long-read assembler (Table 3). The exception to this being Canu, which recovered 100 % of small plasmids in all replicate assemblies. By contrast, most medium (11 of 16; 68 %) and large (4 of 6; 67 %) plasmids were recovered by each long-read-only assembler, with Canu, Raven, Miniasm, and Flye-raw missing plasmids in these categories (Table 2; Fig. 2). Small plasmids are often overlooked but can harbour important virulence and antimicrobial resistance genes and are often present at high copy numbers so can influence gene expression through gene dosage [25]. Further, small plasmids can impose fitness costs similar to that of large plasmids [26] and can be transferred conjugatively, in tandem with larger plasmids, even when missing their own conjugation machinery [27]. It is interesting that plasmid loss primarily occurred in Gram-negative bacteria, most notably *Escherichia coli. E. coli* has been shown to carry cryptic plasmids as small as 1548 bp [28], some of which can harbour antimicrobial resistance genes [29]. Small plasmids found in methicillin-resistant *Staphylococcus aureus* can also harbour antimicrobial resistance genes, thus further supporting the importance of capturing these plasmids during whole-genome assembly [30].

**Table 3.** Summary of plasmids missed by at least one long-read assembler

**Species**	**Isolate**	**Plasmid Size (bp)**	**% Assemblies Missing** **(** ***n*** **=21)**
* Escherichia coli *	ATCC 14028	93 832	10
	ATCC 25922	3173	48
		1919	29
	ATCC 25923	27 490	5
	2021QW-00045	97 090	5
	2021QW-00057	118 339	5
		4715	5
		4084	19
		4063	29
		2101	72
* Staphylococcus aureus *	ATCC 14458	15 773	10
		4439	10
		4326	10
* Klebsiella pneumoniae *	ATCC BAA-2146	117 755	19
		2014	43
	2021QW-00056	95 985	5
* Neisseria gonorrhoeae *	2022 NG-0076	4207	10
	2022 NG-0032	4153	14

Upon closer inspection of recovered plasmids, it was noticed that some long-read-only assemblers occasionally produced multiple copies of a single plasmid and/or erroneously assembled plasmids in the chromosome (Table 2; Fig. 2). This was particularly true for Miniasm and Canu, which produced the largest number of plasmid errors, especially for small plasmids, which were often present as multiple copies (Miniasm: 2 to 9 copies; Canu: 2 to 17 copies) in a single assembly (Table 2; Fig. 2). This issue of multiple plasmid copies in long-read assemblies has been previously described [5]. Flye-raw also produced multiple copies of two medium plasmids in *Klebsiella* isolates 2021QW-00056 and 2020QW-00078 but at much lower frequencies relative to Miniasm and Canu (Table 2; Fig. 2).

**Fig. 3.**
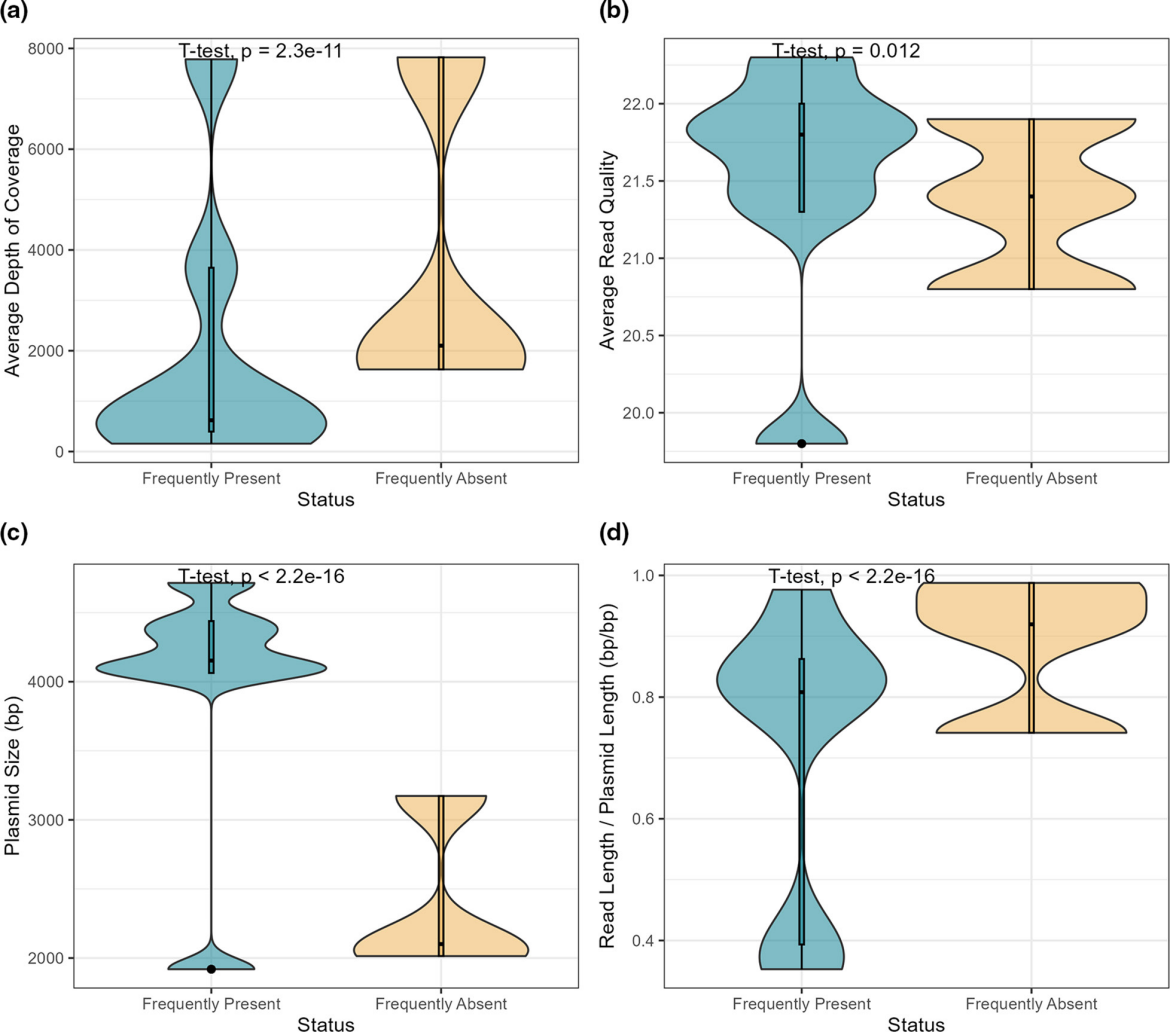
Box-and-violin plots showing the relationship between small plasmid recovery and (a) average long-read depth of coverage, (**b**) average long-read read quality, (**c**) plasmid size, and (d) the ratio between the average long-read length and the length of the associated plasmid. ‘Frequently absent’ plasmids include those not recovered in at least one third of all long-read-only replicate assemblies, whereas ‘frequently present’ plasmids were recovered in at least one third of the replicate assemblies. Significant differences were determined using a two-sided t-test (α=0.05).

The reason for long read assemblers missing small plasmids remains unclear but could be related to differences in sequencing depths and/or read to contig length ratios, as demonstrated in Fig. 3. Small plasmids that were absent in at least one third of all replicate assemblies had significantly greater average read depths (3851±2823 vs 2048±2587; *P*<0.001) and significantly greater average read length to contig length ratios (0.88±0.10 to 0.72±0.23; *P*<0.001), as compared to plasmids that were more frequently recovered (Fig. 3a, d). These plasmids were additionally on average significantly smaller than the other small plasmids (2429±529 bp vs 3957±860 bp; *P*<0.001) (Fig. 3c), thus further supporting the relationship between plasmid recovery and plasmid size. Future studies should continue working to understand why long read assemblers miss small plasmids. Until then, it is recommended that the short-read-first assembler, Unicycler, be used to increase the likelihood that all plasmid sequences are recovered during bacterial genome assembly.

## Conclusion

Results from this study indicate that long-read-only genome assemblers Flye, Miniasm, Canu, and Raven struggle to assemble bacterial plasmids, particularly those smaller than 10 kb. By contrast, the short-read-first assembler, Unicycler, recovered 100 % of plasmids when using Illumina short reads and ONT long reads. As such, it is recommended that Unicycler be used for hybrid genome assembly when plasmid recovery is important.

## Supplementary Data

Supplementary material 1
