# Supplementary material for: Long read genome assemblers struggle with small plasmids
Source: Microb Genom. 2023 May 24;9(5):mgen001024. doi: 10.1099/mgen.0.001024 (PMC10272865; doi:10.1099/mgen.0.001024)
Supplement: Supplementary material 1 [file mgen-9-1024-s001.pdf]

Supplementary Table 1. Long and short read summary statistics and NCBI accession numbers for bacterial isolates used in this study.

Strain	Accession	Genome Size (bp)	Average ONT Read Length (bp)	ONT Read Subset Coverage	Illumina Read Coverage	Predicted Completeness
ATCC 14028	SAMN32977646	4964086	6066	64	57	100.0
ATCC 14458	SAMN32977647	2922051	3521	66	42	99.4
ATCC 23235	SAMN32977648	2747791	3382	73	51	99.5
ATCC 25922	SAMN32977649	5209984	5236	58	82	100.0
ATCC 25923	SAMN32977650	2801053	4164	68	64	99.5
ATCC 33090	SAMN32977651	2887185	5569	62	70	99.5
ATCC BAA-2146	SAMN32977652	5781499	3450	71	107	99.9
2022NG-0032	SAMN33482239	2171594	2823	42	146	99.8
2020QW-00078	SAMN17608176	5563965	10815	51	83	100.0
2021QW-00045	SAMN21919088	5466574	10028	65	64	100.0
2021QW-00056	SAMN21919093	5285754	7145	60	67	100.0
2021QW-00057	SAMN22066472	5215713	7872	68	72	99.6
2022NG-0076	SAMN33482240	2172570	2566	75	139	99.6
2022QW-00133	SAMN32885235	2851245	4242	49	104	99.3

Additional supplementary data and code can be found at https://github.com/johnsonj161/for_publications/tree/107c3dd3865cff8ab5bd86ef70249a65b465aa9c/Long%20read%20genome%20assemblers%20struggle%20with%20small%20plasmids.
